# Promising Highly Targeted Therapies for Cholangiocarcinoma: A Review and Future Perspectives

**DOI:** 10.3390/cancers15143686

**Published:** 2023-07-20

**Authors:** Masaki Kuwatani, Naoya Sakamoto

**Affiliations:** Department of Gastroenterology and Hepatology, Hokkaido University Hospital, North 14, West 5, Kita-ku, Sapporo 060-8648, Japan; sakamoto@med.hokudai.ac.jp

**Keywords:** cholangiocarcinoma, biliary tract cancer, antibody-drug conjugate, photodynamic therapy, photoimmunotherapy

## Abstract

**Simple Summary:**

The challenging early detection of cholangiocarcinoma and the limited availability of less-invasive anticancer therapies contribute to its poor prognosis. Some highly targeted therapies have been explored for cholangiocarcinoma, such as antibody-drug conjugates (ADCs), photodynamic therapy (PDT) with/without systemic chemotherapy, and experimental photoimmunotherapy (PIT). PIT stands out as a novel and promising therapy with less invasiveness; however, it has not yet been performed in human cases of cholangiocarcinoma. In this article, we focus on and review ADC, PDT, and PIT as highly targeted therapies, including experimental therapies for cholangiocarcinoma, and indicate their future prospects.

**Abstract:**

To overcome the poor prognosis of cholangiocarcinoma (CCA), highly targeted therapies, such as antibody-drug conjugates (ADCs), photodynamic therapy (PDT) with/without systemic chemotherapy, and experimental photoimmunotherapy (PIT), have been developed. Three preclinical trials have investigated the use of ADCs targeting specific antigens, namely HER2, MUC1, and glypican-1 (GPC1), for CCA. Trastuzumab emtansine demonstrated higher antiproliferative activity in CCA cells expressing higher levels of HER2. Similarly, “staphylococcal enterotoxin A-MUC1 antibody” and “anti-GPC1 antibody-monomethyl auristatin F” conjugates showed anticancer activity. PDT is effective in areas where appropriate photosensitizers and light coexist. Its mechanism involves photosensitizer excitation and subsequent reactive oxygen species production in cancer cells upon irradiation. Hematoporphyrin derivatives, temoporfin, phthalocyanine-4, talaporfin, and chlorine e6 derivatives have mainly been used clinically and preclinically in bile duct cancer. Currently, new forms of photosensitizers with nanotechnology and novel irradiation catheters are being developed. PIT is the most novel anti-cancer therapy developed in 2011 that selectively kills targeted cancer cells using a unique photosensitizer called “IR700” conjugated with an antibody specific for cancer cells. PIT is currently in the early stages of development for identifying appropriate CCA cell targets and irradiation devices. Future human and artificial intelligence collaboration has potential for overcoming challenges related to identifying universal CCA cell targets. This could pave the way for highly targeted therapies for CCA, such as ADC, PDT, and PIT.

## 1. Introduction

The biliary tree is complicated and finely branched into the liver, which is anatomically classified into small intrahepatic (ϕ20–300 μm), large intrahepatic (300–800 μm), and extrahepatic (>800 μm) bile ducts, including the gallbladder [1]. Its numerical bifurcations can cause difficulty in the detection of and approach to early biliary lesions, especially in the periphery, leading to a poor prognosis of cholangiocarcinoma (CCA), with a 5-year relative survival rate of 7–20% [2,3,4]. To improve prognosis, novel therapeutic approaches to CCA are necessary.

There have been a number of clinical and basic studies on CCA regarding etiology, clinicopathology, molecular biology, and immunology that have clarified the characteristics and targeted points of CCA to some extent. Currently, there are six primary modalities of anticancer treatment for CCA: (1) surgical therapy; (2) chemotherapy; (3) radiotherapy, including brachytherapy and proton radiotherapy; (4) immunotherapy, including immune checkpoint inhibitor and chimeric antigen receptor (CAR) T-cell therapy, (5) phototherapy, and (6) combination therapy, such as antibody-drug conjugate (ADC) therapy (2 + 4), photodynamic therapy (PDT; 2 + 5), and photoimmunotherapy (PIT; 4 + 5). Surgical therapy has developed with the increase in skilled surgeons, high-resolution imaging modalities, and the introduction of portal vein embolization [5]. Chemotherapy has been developed with an understanding of the biology and molecular mechanisms of CCA [6]. Radiotherapy has also improved with internal small, sealed sources [7,8] and proton beams [9], mainly with palliative and supportive intent. Immunotherapy has evolved and improved with the advent of immune checkpoint inhibitors, such as durvalumab (a PD-L1 antibody) [10] and CAR-T therapy [11]. Among them, highly targeted therapies such as ADC, PDT, and PIT are attracting much attention for precision medicine because of their expected high effectiveness and low invasiveness.

To provide highly targeted therapies, previous researchers revealed that CCA abnormally expresses some possible antigens/targets or molecules on the cell surface, such as epidermal growth factor receptor (EGFR), HER2 (c-erbB-2), and vascular endothelial growth factor receptor (VEGFR) [12].

In addition, recent genetic investigations of biliary tract cancer have revealed a range of genetic mutations and alterations specific to the primary cancer site. These include fibroblast growth factor receptor-2 (*FGFR2*) fusion and *IDH1/2* in intrahepatic CCA (ICC); *HER2* (*erbB-2*), *PRKACA*, and *PRKACB* in extrahepatic CCA (ECC: perihilar and distal CCA); *EGFR*, *ERBB3*, and *PTEN* in gallbladder cancer; and *KRAS*, *SMAD4*, and *TP53* shared in ICC and ECC [13,14]. Some of these genetic mutations are also common in pancreatic cancer, which is attributed to the adjacent embryologic relationship between the biliary tree and pancreas [15]. These genetic variations often present challenges and emphasize the importance of implementing precision medicine approaches tailored to the specific cancer site.

Here, we focus on and review three types of highly targeted therapies (ADC, PDT, and PIT), including experimental and promising ones, and indicate their future prospects.

## 2. Antibody-Drug Conjugate

Antibody-drug conjugate (ADC) was developed as a novel drug delivery system for achieving more specific effects on targeted cancer cells and fewer adverse effects on non-targeted normal cells. The first concept of ADC was proposed by Paul Enrlich as a “magic bullet” in 1910 [16], and the first successful conjugation of an antibody and a drug was realized by Mathe et al. in 1958 [17]. The first ADC for malignancy, gemtuzumab ozogamicin, developed for the treatment of acute myeloid leukemia, was approved in 2000 by the US Food and Drug Administration. Since then, 13 other ADCs have been approved for hematological malignancies, such as leukemia and lymphoma (six ADCs), and solid tumors, such as breast cancer and gastric cancer (seven ADCs). Unfortunately, ADCs for CCA have not yet been approved. At present, over 100 ADC candidates have been investigated in clinical stages.

**Figure 1:**
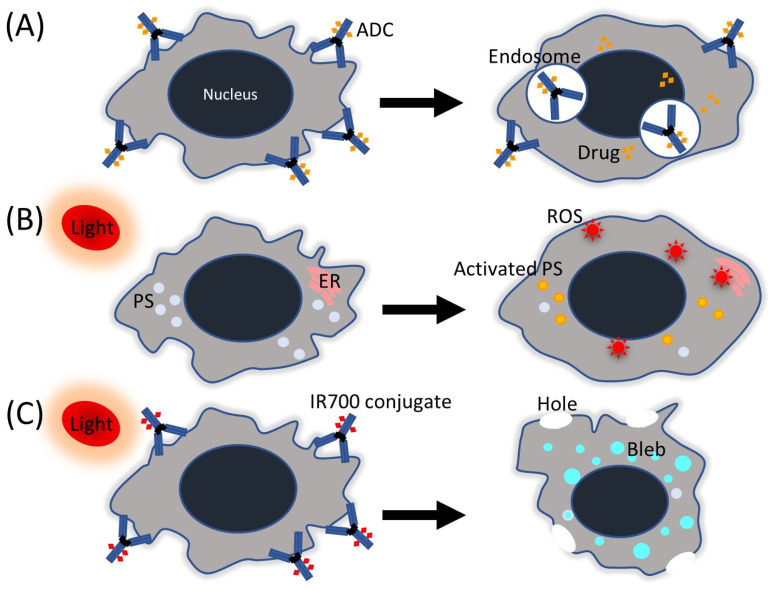
Simple schemas of highly targeted therapies. (**A**) Antibody-drug conjugate (ADC), (**B**) photodynamic therapy (PDT), and (**C**) photoimmunotherapy (PIT). PS, photosensitizer; ER, endoplasmic reticulum; ROS, reactive oxygen species.

ADC comprises: (1) an antibody against targets expressed on tumor cells, (2) a cytotoxic drug called “payload”, and (3) a linker that connects the two. The relative proportions of these three components can vary among ADCs. Among the four subclasses of antibodies, namely IgG1–4, IgG1 is predominantly employed in ADCs due to its extended serum half-life, which is comparable to that of IgG2 and IgG4 (21 days), but longer than that of IgG3 (7–21 days). Additionally, IgG1 exhibits superior complement-fixation and FcγR-binding capabilities compared to the other subclasses [18,19]. FcγR promotes phagocytosis, the release of inflammatory mediators, and antibody-dependent cytotoxicity by effector cells. The mechanism of action of ADCs is more complex than most clinicians believe, often requiring ADC internalization, followed by intracellular processing and payload release (Figure 1A). In detail, the mechanism can be explained in six phases: (1) ADCs circulate as three components. (2) As they diffuse slowly towards target cells, ADCs can release some payload into the tumor microenvironment. (3) Antibody engagement leads to payload-independent antitumor activity via several mechanisms (Fc-mediated stimulation of immune cell effector function and disruption of downstream signaling). (4) Most ADCs are internalized and processed (acidic or proteolytic cleavage of the ADC), largely via antigen-dependent pathways; (5) the payload is released from endosomes and/or lysosomes and causes cell death; and (6) membrane-permeable payloads enter neighboring cells regardless of target expression and can destroy these cells (termed the bystander effect) [20].

ADCs typically employ specific drugs as payloads, such as auristatins (monomethyl auristatin E and monomethyl auristatin F [MMAF]), calicheamicins (ozogamicin), maytansinoids (DM1), and camptothecin analogues (the exatecan derivative, deruxtecan (DXd), and the irinotecan metabolite, SN-38). These payloads exhibit high cytotoxicity at sub-nanomolar concentrations [21]. Early ADCs designed to carry traditional anticancer drugs were not effective compared to the standard anticancer drugs. To enhance the effectiveness of ADCs, the drug-to-antibody ratios of globally approved ADCs range from 2 to 8, on average, depending on the presence of native cysteine or lysine residues on the monoclonal antibody. However, this variation can lead to heterogeneity within a batch of ADCs, presenting a challenge in ADC development [22].

### 2.1. Preclinical Studies of ADCs for CCA

There have been three preclinical trials of ADCs for CCA using murine models whose targeted antigens were human epidermal growth factor receptor 2 (**HER2) [23]**, mucin 1 (**MUC1) [24]**, and **glypican-1 (GPC1) [25]** (Table 1). HER2 is a member of the EGFR family. Protein dimers of HER2 with HER family receptors, such as EGFR, HER2, HER3, or HER4, accelerate cell proliferation and prolong cell survival. Amplification or overexpression of HER2 is predominantly associated with tumorigenesis in the breast (15–30%), stomach, and esophagus (10–30%) [26,27,28]. HER2 overexpression is also observed in 4.8% (95% confidence interval [CI] 0–14.5%) of ICC, 17.4% (95% CI 3.4–31.4%) of ECC, 19.1% (95% CI 11.2–26.8%) of gallbladder cancer, and 27.9% (95% CI 0–60.7%) of ampullary carcinoma [29]. Yamashita-Kashima et al. indicated that the anti-proliferative activity of the ADC trastuzumab emtansine (T-DM1) was higher in CCA cell lines with higher levels of HER2 expression and in proportion to HER2 status [23]. Shinoda et al. showed that the cytotoxicity of lymphokine-activated killer cells (a heterogeneous population consisting of NK, NKT, and T cells) against CCA can be reinforced by staphylococcal enterotoxin A (SEA) conjugated with an antibody directed to MUC1 (MUSE11) in CCA cells [24,30]. SEA is one of the superantigens that bind outside of the peptide-binding groove of MHC class II molecules and activate T cells expressing a certain Vβ type of T cell receptor [31,32]. The transmembrane glycoprotein MUC1 is a mucin family member that can act as a lubricant, moisturizer, and physical barrier in normal cells. MUC1 is also an epithelial mucin antigen that is widely expressed in adenocarcinomas, such as those arising in the pancreas, stomach, ovaries, and bile ducts [33,34,35]. Thus, the SEA-MUSE11 conjugate can work via the above-mentioned phase 4 mechanism alone.

GPC1, a cancer antigen, is a heparan sulfate proteoglycan that is linked to the cell surface by a glycosylphosphatidylinositol anchor and promotes tumor growth, metastasis, and invasion by acting as a co-receptor [36]. Yokota et al. reported high expression of GPC1 in 47% of patients with ECC by immunohistochemical staining and that MMAF-conjugated anti-GPC1 antibodies showed potent tumor growth inhibition against GPC1-positive CCA cells in vitro and in vivo [25]. MMAF is a tubulin-polymerizing inhibitor and is also clinically used as a payload in ADCs for relapsed or refractory multiple myeloma. Therefore, anti-GPC1 ADCs should be clinically investigated and developed, especially for ECC, considering other payloads such as DM1 and DXd.

**CD133/prominin-1** is a pentaspan transmembrane glycoprotein overexpressed in various solid tumors, including colorectal and glioblastomas. In a study by Smith et al. [37], CD133 was found to be highly expressed in ≥50% of pancreatic, gastric, and intrahepatic CCA. In addition, anti-CD133 ADC (maleimidocaproyl-valine-citrulline-p-aminobenzoyl-MMAF [vcMMAF]) treatment resulted in a significant delay in Hep3B (hepatocellular carcinoma) tumor growth in SCID mice. Thus, the anti-CD133 antibody-vcMMAF conjugate could also be effective for CCA treatment.

### 2.2. Human Studies of ADCs for CCA

Seven ADCs have been approved for solid tumors worldwide. Among these, three are approved for breast cancer targeting HER2, two for gastric cancer targeting HER2, and one each for head and neck cancers targeting EGFR/tissue factor and urothelial cancer targeting nectin-4 (including duplicate indications). In addition, there have been three clinical trials specifically focused on CCA (Table 1). Two of them target **HER2**, and one targets **FGFR2** on CCA cells.

Mondaca et al. showed one case with a 30% partial response of the primary lesion after trastuzumab (anti-**HER2** antibody)-DM1 treatment [38]. Additionally, Tsurutani et al. demonstrated confirmed objective responses in HER2-expressing (IHC ≥1+) non-small cell lung cancer, salivary gland cancer, endometrial cancer, and biliary tract cancer following trastuzumab-DXd treatment. Among these cases, two showed tumor shrinkage of ≥60% [39]. Based on its promising results, a multicenter phase II trial of trastuzumab deruxtecan for HER2-positive unresectable or recurrent biliary tract cancer (HERB trial) is ongoing in Japan [40].

To target **FGFR2**, aprutumab ixadotin (BAY 1187982) was developed as the first ADC with a novel auristatin-based payload. A phase I trial comprising 20 patients with FGFR2-positive solid tumors, including four CCAs, revealed that aprutumab ixadotin was poorly tolerated due to a high rate of proteinuria and nephropathy. The maximum tolerance dose (MTD) was found to be below the therapeutic threshold estimated preclinically; therefore, the trial was terminated early [41]. The authors hypothesized that severe toxicity might be attributed to the unique combination of an auristatin W derivative payload with aprutumab. As FGFR2 fusion/rearrangement is detected in 7.4−13.6% of ICCs, improvement of anti-FGFR2 antibody-based ADC is also warranted [42,43,44].

## 3. Photodynamic Therapy

Since the ancient Egyptian era, light has been used in medicine to treat skin diseases such as psoriasis and vitiligo with psoralens. At present, photodynamic therapy (PDT) is extensively used in infectiology, dermatology, gynecology, urology, and oncology [45]. Since the first modern medical concept of photodynamic therapy was introduced in 1900 by Raab and von Tappeiner based on the incidental effect on malaria-causing protozoa, the first-in-human PDT of tumors was performed by von Tappeiner for skin tumors in 1903, according to the literature [45,46,47,48]. Approximately 70 years later, Diamond et al. published a groundbreaking report demonstrating the anticancer effect of PDT with hematoporphyrin and white light on rat gliomas in both in vitro and in vivo settings [49]. Notably, the PDT effect was limited to the area where hematoporphyrin and white light coexisted, thus achieving highly targeted treatment. The mechanism of PDT is explained by the following phases: (1) entering the cell of the photosensitizer; (2) photoexcitation of the photosensitizer from the ground energy state to the exited state; (3) energy transfer from the photosensitizer to the biomolecules from its surroundings (type I reaction) or to the oxygen molecule (type II reaction); (4) production of reactive oxygen species (ROS): superoxide anion radical (O_2_^•−^), hydroxyl radical (OH^•^) by type II, and singlet oxygen (^1^O_2_) by type I inside the cells; and (5) oxidative stress resulting in the destruction of cancer cells [50,51] (Figure 1B). Furthermore, recent studies indicate that immunogenic cell death via apoptosis and necroptosis can occur after PDT, leading to the release of danger/damage-associated molecular patterns that can activate an adaptive immune response [52,53].

In 1976, Kelly and Snell conducted the first human study to evaluate the effects of PDT using a hematoporphyrin derivative (HpD) in five patients with bladder cancer. Their study resulted in one successful case, demonstrating the potential of PDT in cancer treatment [54]. Nowadays, PDT has been approved for the treatment of various types of cancer, including head and neck, brain, lung, esophagus, breast, prostate, bladder, skin, pancreas, and bile duct cancers [55]. Many researchers have made efforts to improve the efficacy of PDT through the refinement of photosensitizers and light sources, as described below.

### 3.1. Photosensitizers

Since the initial report of topical eosin combined with white light for treating skin tumors in 1903, various photosensitizers have been developed to enhance the efficacy of PDT (Table 2). A significant breakthrough occurred in 1942 and 1948 when porphyrins, including hematoporphyrin, coproporphyrin, protoporphyrin, and zinc hematoporphyrin, were found to selectively accumulate in malignant tumors after their exogenous administration for tumor detection experiments [56,57]. Among the porphyrins, HpD was identified as the most phototoxic for killing tumors in mice. In a pivotal study conducted by Dougherty et al. (1975), HpD demonstrated a high antitumor effect of PDT in vivo, while HpD alone or light alone had no effect [58]. In 1976, Kelly et al. indicated the effects of PDT using HpD in five patients with bladder cancer, as described above [54].

Since then, various photosensitizers have been developed. The second generation ones (hematoporphyrin derivatives and synthetic photosensitizers, such as 5-aminolevulinic acid, benzoporphyrin derivatives, texaphyrins, thiopurine derivatives, chlorin, bacteriochlorin analogues, and phthalocyanines) were developed to address the drawbacks of the first-generation ones, such as low chemical purity and poor tissue penetration due to maximum absorption at a relatively short wavelength (630 nm). The third-generation photosensitizers comprise second-generation photosensitizers combined with molecules/LDL and lipoproteins/antibodies specifically targeted to antigens. These approaches increase the affinity and permeability of cancer cells and lower skin toxicity. The first- and second-generation photosensitizers are also classified among the porphyrin family (HpD, benzoporphyrin derivative, ALA [5-aminolevulinic acid]), chlorin family (temoporfin, purlytin [tin-ethyl-etiopurpurin], NPe6 [mono-L-aspartyl chlorin e6] = talaporfin sodium, HPPH (photochlor), and dyes (phthalocyanine and naphthalocyanine) based on their chemical structures [50]. Among them, HpD, temoporfin (m-THPC), phthalocyanine-4, talaporfin, and chlorine e6 derivatives (including chlorin I/chlorin II) have mainly been used clinically and preclinically for CCA/bile duct cancer [59,60,61,62,63,64,65,66,67,68]. In vitro studies have shown varying degrees of efficacy of these photosensitizers against human CCA cells, and similar results have been observed in animal studies. Phthalocyanine and chlorin I/chlorin II have not yet been clinically investigated in patients with CCA.

Among the third-generation photosensitizers, nanoparticle albumin-bound (nab)-mTHPC, which is produced using nanotechnology, showed high cytotoxicity in a CCA cell line called TFK-1 when subjected to illumination. Notably, no toxicity was observed under dark conditions [69]. Furthermore, the use of interstitially targeted liposomes containing metalated phthalocyanines, specifically targeting tumor cells and the tumor microenvironment, has shown promising results. These photodynamically active photosensitizers were able to effectively photosensitize human CCA cells (SK-ChA-1) and non-cancerous cells (human endothelial cells [HUVECs], murine fibroblasts [NIH-3T3], and murine macrophages [RAW 264.7]) [70]. Thus, future animal and clinical studies exploring their efficacy are warranted.

### 3.2. Light Sources for PDT

Light sources for PDT have evolved over time. Initially, non-laser light sources such as conventional lamps were used, which involved water-cooled incandescent lamps with output defined by filters. However, these light sources had limitations, such as a significant thermal component, making it challenging to accurately calculate the delivered light dose [71]. In recent years, there has been a shift towards more efficient lasers that can produce monochromatic light of a known wavelength and easily perform light dosimetry. Laser light can also be supplied via an optical fiber for localized treatment. The appropriate wavelength for PDT is determined according to the photosensitizer used: 630 nm for HpD, 635 nm for 5-ALA-induced protoporphyrin IX, and 652 nm for tetra (m-hydroxyphenyl) chlorin (Table 2). Moreover, recent advances in semiconductor diode technology have enabled laser systems to be more compact and cheaper [45]. Thus, at present, lasers might be the first choice for a PDT light source. Meanwhile, it has been reported that the use of light emitting diodes (LEDs) of specific wavelengths (610 nm) is effective for PDT of CCA [72,73] and gastrointestinal cancers [74]. Therefore, LEDs can be another light source because of their smaller batteries and lower cost compared to conventional lasers.

### 3.3. Human Clinical Studies on PDT

Despite ingenuity and improvements in the above-mentioned modalities, PDT monotherapy has limitations, such as incomplete tumor killing or recurrence, and has not replaced existing antitumor therapies. In the field of biliary tract cancer, PDTs mainly for ECC have been clinically performed worldwide since the 1990s (Table 2) [75,76,77]. However, since the 2000s, some RCTs and one meta-analysis have revealed that PDT could yield significantly longer survival/higher survival rates than supportive care with biliary drainage [78,79,80,81,82,83,84]. The 1-year survival rate of the PDT with stent group was 56% (range: 39–75%), and that of the control group was 25% (range: 12–38%). The 2-year survival rate of the PDT with stent group was 16% (range: 14–21%), and that of the control group was 7% (range: 3–10%). Meanwhile, PDT has not shown superiority over conventional systemic chemotherapy in many clinical trials [85,86].

Alternatively, some PDTs combined with systemic chemotherapy (chemophototherapy, CPT) have been shown to be effective treatment options preclinically and clinically [86,87]. During in vitro and in vivo experiments, PDT with novel cancer drugs, such as DMXAA (ASA404: flavone acetic acid analog with TNF-α synthesis), PD166285 (synthetic RTK inhibitors), and TNP-470 (synthetic anti-angiogenesis agent), has demonstrated good outcomes for murine sarcoma, breast, colon, and prostate cancer cells, although CCA cells were not included [85].

There have been several clinical comparative studies that have examined the effectiveness of PDT vs. CPT or chemotherapy vs. CPT for CCA. [86,87]. Regarding PDT vs. CPT, two studies, including one RCT, revealed that CPT had superior outcomes (median overall survival: 8 M vs. 17 M, *p* = 0.005; 12 M vs. 17 M, *p* = 0.021), while two studies indicated the equivalence of both treatments (11 M vs. 18 M, *p* = 0.05; 15 M vs. 20 M, *p* = 0.727) [87]. Meanwhile, according to a meta-analysis of chemotherapy versus CPT or PDT vs. CPT, CPT had significantly better overall survival than chemotherapy or PDT alone (CPT vs. chemotherapy, hazard ratio (HR): 0.69, *p* = 0.02; CPT vs. PDT, HR: 0.36, *p* < 0.01) [86]. Therefore, for unresectable CCA, PDT with systemic chemotherapy (CPT) is expected to yield the most favorable outcomes, although the standard PDT or combined chemotherapy regimen is yet to be determined.

**Table 2:** Photosensitizers for photodynamic therapy and photoimmunotherapy.

			Investigations for CCA					
Photosensitizer	Potential Indications	Activation Wavelength	Human	Animal	In Vitro	LED Effect	LED Wavelength	Reports Regarding CCA *
**Photodynamic therapy (PDT)**								
HPD (partially purified) porfimer sodium	Cervical, endobronchial, oesophageal, bladder, gastric cancers, brain tumor, **bile duct cancer**	630 nm	✓	✓	✓	✓	620–650 nm	[60,61,75,76,77,78,79,80,81,82,83]
Phosphorus tetraphenylporphyrin	**Bile duct cancer**	610 nm			✓	✓	610 nm	[72,73,74]
m-THPC (Temoporfin)	Head and neck, prostate, pancreas, lung, skin, **bile duct cancer**	652 nm	✓	✓	✓			[62,75]
Phthalocyanine-4	Cutaneous/subcutaneous lesions from diverse solid tumor origins, **bile duct cancer**	670 nm		✓	✓			[63]
Taporfin sodium (Talaporfin, NPe6)	Lung, liver metastasis, pancreas, colon, brain cancer, **bile duct cancer**, solid tumors from diverse origins	664 nm	✓	✓	✓	✓	660 nm	[64,65,66,73]
Chlorine e6 derivatives	Nasopharyngeal, sarcoma, brain, pancreatico**biliary** malignancies	660 nm	✓	✓	✓			[67,68,75]
Chlorin I/chlorin II	**Bile duct cancer**	650 nm		✓	✓			[69]
Nanoparticle albumin-bound mTHPC	**Bile duct cancer**	652 nm			✓			[70]
ITLs encapsulating ZnPC and AlPC	Breast, **bile duct cancer**	671 nm			✓			[71]
5-ALA	Basal-cell carcinoma, head and neck, gynaecological tumors brain, bladder tumors	635 nm 375–400 nm						
5-ALA-methylesther	Basal-cell carcinoma	635 nm						
5-ALA benzylesther	Gastrointestinal cancer	635 nm						
5-ALA hexylesther	Bladder tumors	375–400 nm						
Boronated protoporphyrin	Brain tumors	630 nm						
BPD-MA (benzoporphyrin)	Basal-cell carcinoma	689 nm						
HPPH	Basal-cell carcinoma, head and neck, esophagus, lung cancer	665 nm						
Lutetium texaphyrin	Cervical, prostate and brain tumours	732 nm						
Motexafin lutetium (Mlu)	Prostate cancer	732 nm						
Padeliporfin	Prostate cancer	762 nm						
Redaporfin	Head and neck cancer	749 nm						
Silicon phthalocyanine	Cutaneous T-cell lymphoma	675 nm						
SnET2	Cutaneous metastatic breast cancer, basal-cell carcinoma, Kaposi’s sarcoma, prostate cancer	664 nm						
Verteporfin	Skin, pancreas cancer	690 nm						
**Photoimmunotherapy (PIT)**								
IR700 (IRDye 700DX N-hydroxysuccinimide ester)	Head and neck can, stomach, esophagus, pancreas, **bile duct cancer**	690 nm		✓	✓	✓	680–700 nm	[88,89]

CCA—cholangiocarcinoma; LED—light emitting diode; HPD—hematoporphyrin derivative; m-THPC—metatetrahydroxyphenylchlorin = temoporfin; ITLs—Interstitially targeted liposomes; ZnPC—zinc phthalocyanine; AIPC—aluminum phthalocyanine; 5-ALA—5-aminolevulinic acid; BPD-MA—benzoporphyrin derivative-monoacid ring A; HPPH—2-(1-hexyloxyethyl)-2-devinyl pyropheophorbide-alpha; SnET2—tin ethyl etiopurpurin; * Brackets include reference numbers. ✓ indicates the existence of previous reports.

## 4. Photoimmunotherapy

Photoimmunotherapy (PIT) is a novel anti-cancer therapy developed in 2011 by Kobayashi H. et al. that theoretically selectively kills targeted cancer cells with no damage to normal cells [90]. PIT comprises a unique photosensitizer called “IR700.” This water-soluble photo dye consists of silicon phthalocyanine and hydrophilic side chains (IRDye 700DX N-hydroxysuccinimide ester) (Table 2). In addition, PIT incorporates a specific molecule that enables binding to a target cell, which can be an antibody or a low-molecular-weight compound. Examples include the combination of IR700 with an anti-EGFR antibody or an affibody combined with IR700 as antibody/affibody-photosensitizer conjugates (APCs) [91,92]. IR700 is different from photosensitizers used in PDT because IR700 has a more than five-fold higher extinction coefficient (2.1 × 10^5^ M^−1^cm^−1^ at the absorption maximum of 689 nm) [93] than that of conventional photosensitizers, such as the HpD (1.2 × 10^3^ M^−1^cm^−1^ at 630 nm), meta-tetrahydroxyphenylchlorin (2.2 × 10^4^ M^−1^cm^−1^ at 652 nm), and mono–L-aspartylchlorin e6 NPe6 (4.0 × 10^4^ M^−1^cm^−1^ at 654 nm) [94]. Although PIT also requires a light source emitting near-infrared light (NIR) with a wavelength of 689–700 nm (NIR wavelength is usually defined as 650–1700 nm) [90,95], its mechanism differs from that of PDT (Figure 1C). In PIT, the destruction of the target cell starts with a chemical change of IR700 in APCs bound to the cell due to the release of hydrophilic side chains of IR700 after NIR irradiation. This process forms water-insoluble aggregates of APCs or APC-antigen complexes on the cell surface molecules, leading to physicochemical changes within the APC-antigen complex that reduce cell membrane integrity because of damage to transmembrane target proteins. Subsequently, water flows into the cytoplasm, causing cell swelling [96]. In contrast, the mechanism of PDT is based on inner cell destruction by ROS (Figure 1B). In addition, PIT also results in further immunogenic cell death that is initiated by the maturation of immature dendritic cells with released tumor antigens from the treated cancer cell in the adjacent microenvironment. After cancer cell-targeted PIT, CD8+ T cells newly primed by a larger repertoire of cancer antigens proliferate in the treated tumor beds. Finally, anticancer host immunity can be strengthened after cancer-cell-targeted PIT [96]. Although many investigations of PIT and clinical PIT for head and neck cancer use lasers as an NIR light source as well as PDT, LEDs can also produce NIR and yield a PIT effect on CCA cells [88]. As described above, CCA can develop in the extrahepatic and intrahepatic bile ducts that cannot be approached/visualized by an endoscope, while a special catheter with LEDs, such as a biliary drainage tube, can be placed for PIT at the peripheral CCA [88]. Therefore, a dedicated PIT device for CCA would shed light on PIT for CCA.

### 4.1. Antibody and Low Molecular Weight (LMW) Compound for Targeting Cancer Cells with IR700

IR700 can covalently bind to any kind of antibody (IR700:antibody = approximately 3:1), forming a highly flexible theranostic platform [90]. The selected antibody or low-weight molecule for PIT depends on the cell surface antigen or molecules on the target cancer cell. Various agents, including many APCs and some LMW compound-IR700 conjugates, have been reported in preclinical and clinical settings. EGFR is a representative target on cancer cells for antibodies, followed by HER2, carcinoembryonic antigen (CEA), VEGFR2, cadherin-17, and ICAM-1 in PIT [97]. In addition to the antibody, a partner of IR700 for a conjugate of PIT that has been reported so far includes an affibody (a small protein mimetic [6–7 kDa]) for HER2-overexpressing breast cancer [92], an LMW ligand composed of Glu-Urea-Lys for prostate cancer with prostate-specific membrane antigen [98], and a lection for CEA-expressing pancreatic cancer [99,100]. For CEA, a novel approach with affimer proteins and cubosomes has also been reported in colorectal cancer cell lines [89]. For PIT of CCA, there have been two preclinical reports: one used anti-**EGFR** and **HER2** antibodies [88], while another used an antibody against **trophoblast cell surface antigen 2**/**tumor-associated calcium signal transducer 2 (TROP2)**, which is overexpressed in trophoblast cancer and many epithelial cancers, including pancreatic cancer and CCA [101]. In both studies, PIT specifically killed the target-expressing CCA cells in vitro and significantly suppressed the growth of CCA in murine xenograft models after NIR irradiation.

### 4.2. Human Clinical Studies on PIT

In 2020, PIT for unresectable locally advanced/recurrent head and neck cancer was publicly approved and initiated as a clinical modality in Japan ahead of the rest of the world. In the U.S., a phase 3 trial of PIT for the same cancer has been performed. The first in-human PIT for head and neck cancer conducted in the U.S. in 2015 revealed tumor volume reduction with complete remission (13.3%), partial remission (30%), and stable disease (36.7%), which is a promising but somewhat insufficient result [102]. Thereafter, in Japan, a few clinical trials have been performed or initiated for unresectable advanced/recurrent gastric cancer or esophageal cancer (one for gastric cancer or esophageal cancer is completed; another for esophageal cancer is suspended) and advanced or recurrent solid tumors with one or more hepatic metastases (recruitment) (https://jrct.niph.go.jp/search?language=en&page=1, accessed on 1 June 2023). The former used cetuximab-IR700 conjugate targeting cancer cells with anti-PD antibody (nivolumab), while the latter used anti-CD25 antibody-IR700 conjugate targeting regulatory T-cells with anti-CTLA-4 antibody (pembrolizumab). The initial PIT with an APC on a cancer cell alone has some limitations, such as (1) insufficient attainment of an APC to a cancer cell and penetration depth of NIR light into the tissue; (2) lack of a dedicated light device for a deeply located tumor, such as CCA and pancreatic cancer; and (3) non-universality of a single APC for various tumors. Based on these findings, PIT with another anticancer drug, such as an immune check inhibitor (ICI), is ongoing, as described above. Unfortunately, there has been no clinical trial of PIT for CCA. However, recent molecular and immunological investigations and treatments for CCA, including EGFR and FGFR inhibitors (pemigatinib/infigratinib/futibatinib), neurotrophic receptor tyrosine kinase inhibitor (entrectinib/larotrectinib), and ICIs (durvalumab, a PD-L1 inhibitor), will promote and accelerate the use of PIT in the near future.

## 5. Conclusions and Future Directions

The basic premises of highly targeted therapies for CCA involve the identification of specific targets on CCA cells and directing therapeutic interventions towards those targets. Although several targets have been identified or investigated as described above, they are not universally applicable and are limited to certain types of CCA. Overcoming the discrepancy between the universality of the target and the localization of the attack in cancer therapy is partially controversial. However, it is not an insurmountable challenge, as we can visually differentiate CCA cells from non-CCA cells and select them in vitro using our own judgment. Currently, artificial intelligence (AI) plays an important role in various fields, including clinical diagnosis of cancer through endoscopy and pathology, as well as preclinical analysis using multi-omics approaches [103]. Therefore, the collaboration between human observation and AI has the potential to overcome the obstacles in identifying universal targets in CCA cells, paving the way for the development of highly targeted therapies for CCA, such as in ADC, PDT, and PIT.

## Figures and Tables

**Table 1:** Targets for cholangiocarcinoma cells.

		Preclinical Study			Clinical Study	
Name	Type	ADC	ADC-Payload	PIT	ADC	ADC-Payload
HER2	Receptor	✓	Emtansine (DM1)	✓	✓	Emtansine (DM1) Deruxtecan (DXd)
EGFR	Receptor			✓		
FGFR2	Receptor				✓	Ixadotin
MUC1	Secretion (mucin)	✓				
Glypican-1 (GPC1)	Secretion (proteoglycan)	✓	Monomethyl auristatin F (MMAF)			
CD133/ prominin-1	Secretion (glycoprotein)	✓	Maleimidocaproyl-valine-citrulline-p-aminobenzoyl-MMAF (vcMMAF)			
TROP2	Secretion (glycoprotein)			✓		

HER2—human epidermal growth factor receptor 2; EGFR—epidermal growth factor receptor; FGFR2—fibroblast growth factor receptor-2; MUC1—transmembrane glycoprotein mucin 1; TROP2—trophoblast cell surface antigen 2. ✓ indicates the existence of previous reports.
